# In silico pathway analysis in cervical carcinoma reveals potential new targets for treatment

**DOI:** 10.18632/oncotarget.6667

**Published:** 2015-12-19

**Authors:** Peter A. van Dam, Pieter-Jan H. H. van Dam, Christian Rolfo, Marco Giallombardo, Christophe van Berckelaer, Xuan Bich Trinh, Sevilay Altintas, Manon Huizing, Kostas Papadimitriou, Wiebren A. A. Tjalma, Steven van Laere

**Affiliations:** ^1^ Antwerp University Hospital, Centre of Oncologic Research (CORE) Antwerp University, Edegem, Belgium; ^2^ Multidisciplinary Oncologic Centre Antwerp (MOCA), Edegem, Belgium; ^3^ Phase I - Early Clinical Trials Unit, Oncology Department, University Hospital Antwerp UZA, Edegem, Belgium

**Keywords:** cervical carcinoma, cancer, in silico pathway analysis, treatment targets

## Abstract

An in silico pathway analysis was performed in order to improve current knowledge on the molecular drivers of cervical cancer and detect potential targets for treatment. Three publicly available Affymetrix gene expression data-sets (GSE5787, GSE7803, GSE9750) were retrieved, vouching for a total of 9 cervical cancer cell lines (CCCLs), 39 normal cervical samples, 7 CIN3 samples and 111 cervical cancer samples (CCSs). Predication analysis of microarrays was performed in the Affymetrix sets to identify cervical cancer biomarkers. To select cancer cell-specific genes the CCSs were compared to the CCCLs. Validated genes were submitted to a gene set enrichment analysis (GSEA) and Expression2Kinases (E2K). In the CCSs a total of 1,547 probe sets were identified that were overexpressed (FDR < 0.1). Comparing to CCCLs 560 probe sets (481 unique genes) had a cancer cell-specific expression profile, and 315 of these genes (65%) were validated. GSEA identified 5 cancer hallmarks enriched in CCSs (*P* < 0.01 and FDR < 0.25) showing that deregulation of the cell cycle is a major component of cervical cancer biology. E2K identified a protein-protein interaction (PPI) network of 162 nodes (including 20 drugable kinases) and 1626 edges. This PPI-network consists of 5 signaling modules associated with MYC signaling (Module 1), cell cycle deregulation (Module 2), TGFβ-signaling (Module 3), MAPK signaling (Module 4) and chromatin modeling (Module 5). Potential targets for treatment which could be identified were CDK1, CDK2, ABL1, ATM, AKT1, MAPK1, MAPK3 among others. The present study identified important driver pathways in cervical carcinogenesis which should be assessed for their potential therapeutic drugability.

## INTRODUCTION

Cervical cancer is the second most prevalent cancer seen in woman worldwide, with about 500.000 cases and over 270.000 deaths estimated annually [1]. The etiological role of infection with high-risk papilloma viruses in cervical carcinoma is well established [2]. However, little is known on the regulatory networks of biological factors involved in cervical cancer. Somatic mutations in PIK3CA, PTEN, TP53, STK11, KRAS, MAPK1, HLA-B, EP300, FBXW7, NFE2L2, TP53, ERBB2, as well as several copy number alterations have been implicated in the pathogenesis of cervical carcinomas [3, 4]. Over the last decades little progress has been made in the systemic treatment of patients with advanced or recurrent cervical cancer [5]. In the current study we designed an in silico approach to identify potential driver pathways of cervical carcinogenesis and candidate targets for treatment. Three publicly available Affymetrix expression datasets were integrated, allowing us to interrogate the molecular profile of the malignant transformation of normal cervical tissue into high-grade intraepithelial neoplasia (HSIL) and then into invasive cervical cancer (ICC) using the largest in silico series of cervical samples ever reported. Gene set enrichment analysis (GSEA) enabled us to unravel cervical cancer biology and Expression2Kinases (E2K) to delineate the driving signaling network. A PPI-network consisting of 5 signaling nodes was identified.

## RESULTS

### Differential gene expression analysis

**Figure 1:**
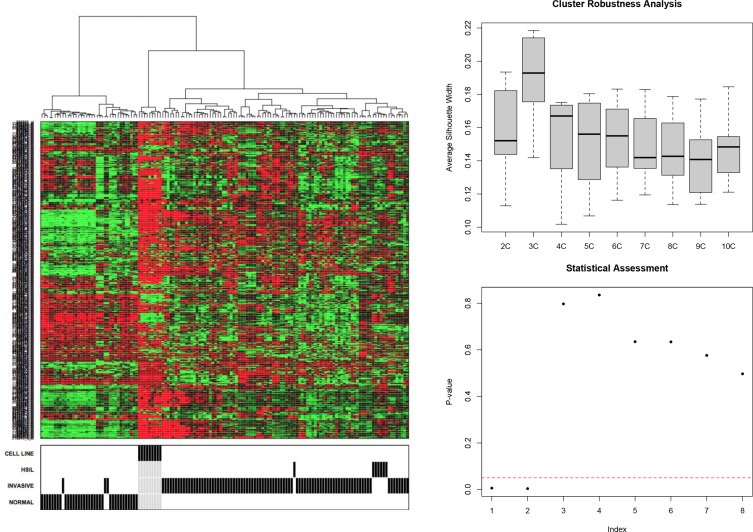
Unsupervised hierarchical cluster analysis (1A) and cluster robustness analysis was performed (1B–1C)

**Figure 2:**
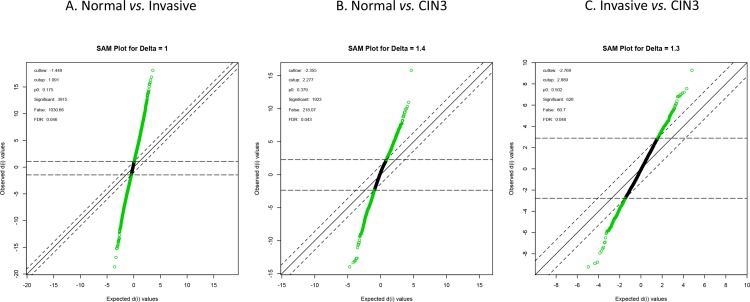
Differential gene expression analysis

An unsupervised hierarchical cluster analysis on publicly available expression data of normal cervical samples, high grade intraepithelial neoplasia (CIN3) samples, cervical cancer samples and cervical cancer cell lines was performed and cluster robustness analysis showed that the optimal result consists of 3 clearly separating groups (Figure 1): the normal samples, the cell lines and the invasive cancer samples that lumped together with the CIN3 samples. Differential gene expression analysis with a false discovery rate smaller than 5% identified 3,915 genes that were differentially expressed between the normal samples and the invasive carcinomas, 1,923 genes between the normal samples and the CIN III lesions and 628 genes between the CIN III and the invasive carcinomas (Figure 2A–2C). We next wanted to identify cervical cancer cell intrinsic expression patterns. Therefore, we evaluated only the expression levels of the probe sets which were overexpressed in the cervical invasive cancer samples compared to the normals cervical samples (*N* = 1,547/3,915) in a collection of cervical cancer cell lines. Of these, 729 probe sets were differentially expressed between the cervical invasive cancer samples and the cell lines: 560 probe sets (481 unique genes) were overexpressed in the cell lines (i.e. cancer cell-related expression) and 169 probe sets (133 unique genes) were repressed in the cell lines (i.e. potentially stroma-related expression). The set of 481 genes was retained for further data analysis.

### Validation of the data

**Figure 3:**
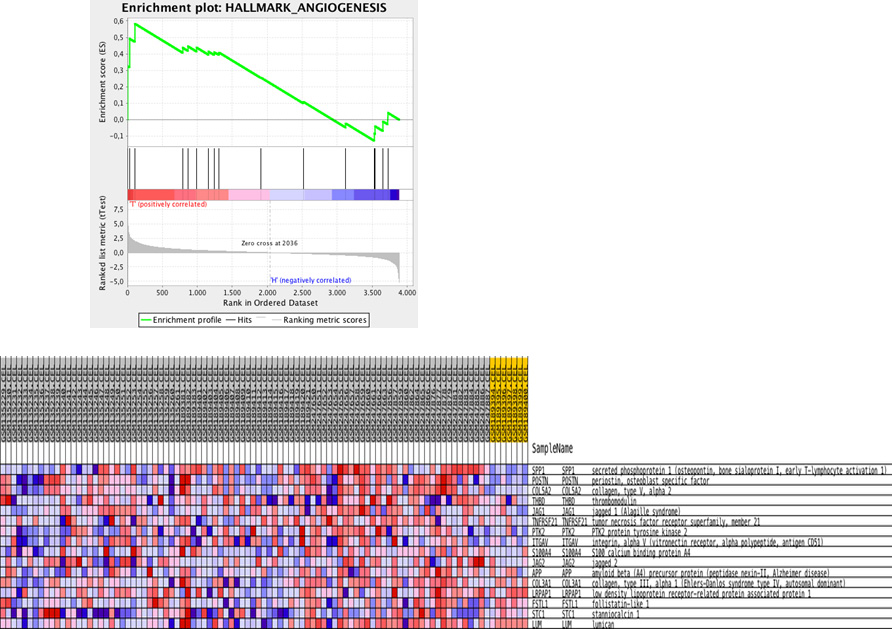
Gene set enrichment analysis for KEGG pathways mapping showing enrichment plot on the hallmark Angiogenesis comparing normal with invasive cancer samples

Data validation was done using two alternative strategies. First, to assess whether the data are biologically relevant we evaluated the expression profiles of genes implicated in angiogenesis and vessel maturation, as we and several other groups have reported that during the progression from normal to noninvasive, and then to microinvasive cervical carcinoma the microvessel density increases significantly [6–9] We found that gene expression profiles of CIN3 samples, when compared to the normal cervical samples were enriched for angiogenic genes. Similar enrichment results were observed in gene expression profiles of cervical cancer samples when comparing them to CIN3 or normal samples. Figure 3 shows a gene set enrichment plot comparing normal cervical samples to invasive cervical cancer samples for genes associated to “Angiogenesis” according to the molecular signatures database “Hallmark” category. In a second validation strategy, we evaluated the expression levels of the list of 481 genes overexpressed in cervical cancer cell lines relative to cervical cancer samples, thus putative cervical cancer cell intrinsic genes, in an independent data set (Agilent GSE7410). In total, 315 (66%) putative biomarkers for cervical cancer were validated. The top 10 genes by fold-change consisted of TK1, UBE2C, KIAA0101, FANCI, TYMS, CDK1, RRM2, CENPF, PTTG1 and KNTC1.

### Pathway analysis

**Figure 4:**
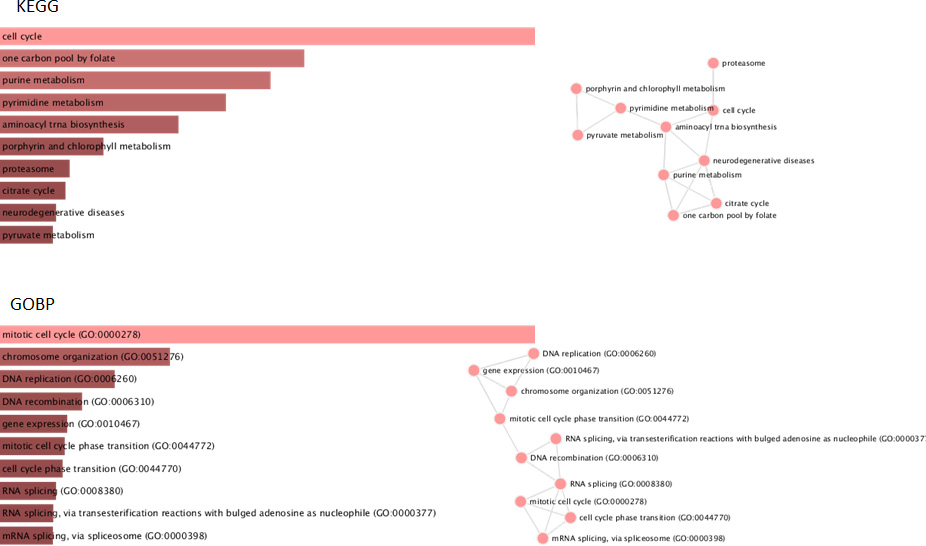
Gene Set Enrichment Analysis

Using the set of validated genes overexpressed in cervical cancer samples relative to normal cervical samples (*N* = 315), gene set enrichment analysis (GSEA) for pathways contained in the Kyoto Encyclopedia of Genes and Genomes (KEGG) database was performed. In addition, similar analyses were done for Gene Ontology gene sets associated with biological processes (GOBP). Barplots and interaction networks for the top 10 most significant hits for each database are summarized in Figure 4. These results reveal that the list of 315 cervical cancer intrinsic genes are mostly involved in the cell cycle by means of processes like DNA replication and recombination, RNA metabolism, purine and pyrimidine metabolism.

**Figure 5:**
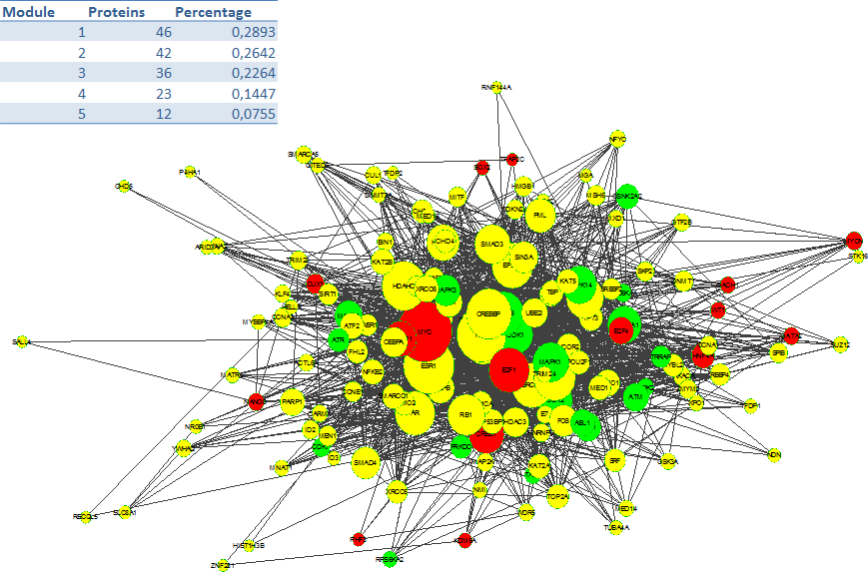
PPI Network (E: 1620; N: 162)

**Figure 6:**
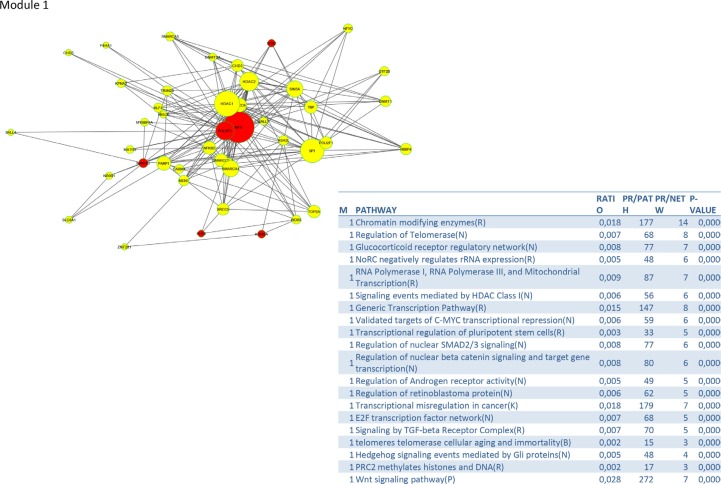
PPI-network associated with MYC signaling

**Figure 7:**
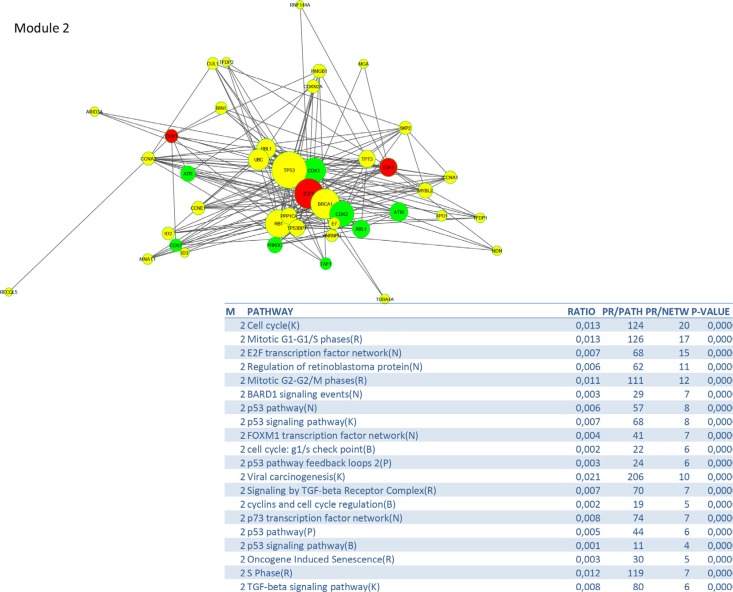
PPI-network related to cell cycle deregulation

**Figure 8:**
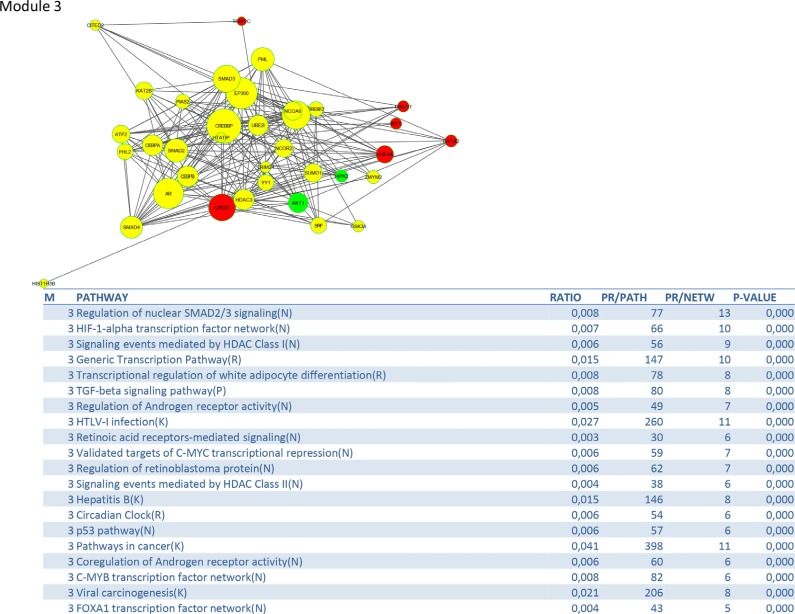
PPI-network related to TGFβ-signaling

**Figure 9:**
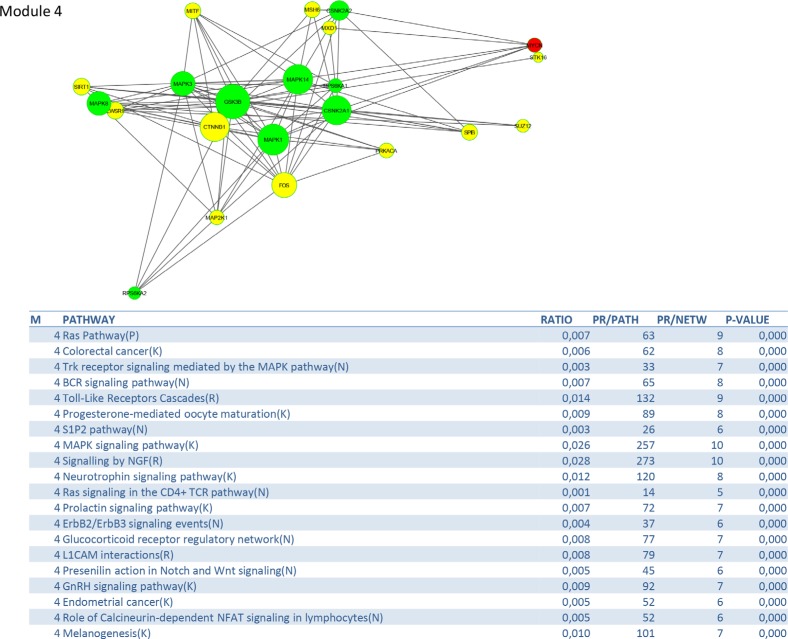
PPI-network related to MAPK signaling

**Figure 10:**
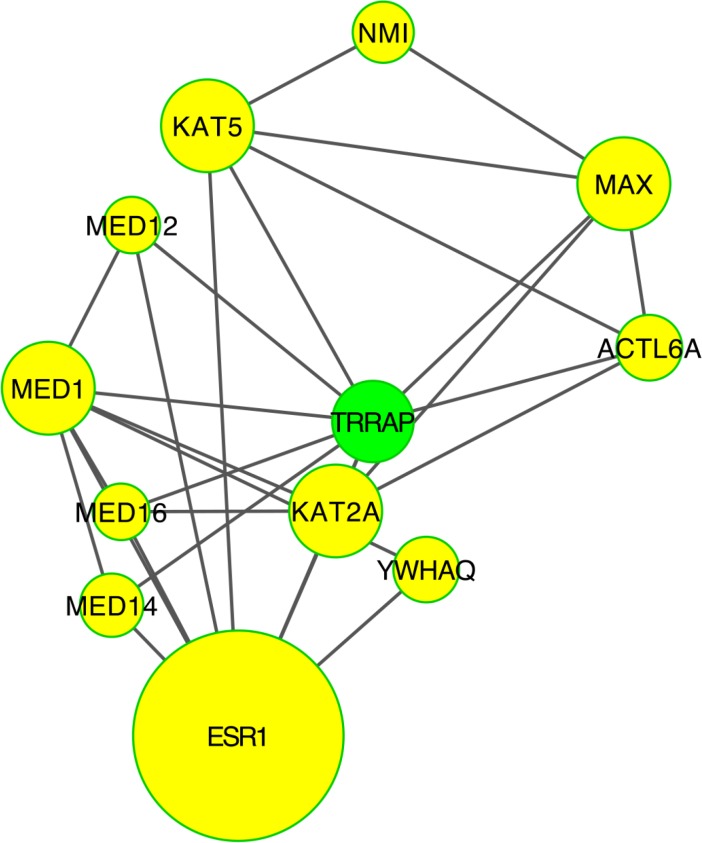
PPI-network related to chromatine remodeling Number of genes involved in this module was to small to perform a meaningful pathway analysis.

To identify signalling pathways responsible for driving the observed gene expression differences and thus the cell cycle related changes, E2K was performed. A protein-protein interaction (PPI) network of 162 nodes (including 20 druggable kinases) and 1626 edges was identified (Figure 5). This PPI-network consists of 5 signaling modules associated with MYC signaling (Module 1 – Figure 6), cell cycle deregulation (Module 2 – Figure 7), TGFβ-signaling (Module 3 – Figure 8), MAPK signaling (Module 4 – Figure 9) and chromatine modeling (Module 5- Figure 10). Potential targets for treatment that could be identified were CDK1, CDK2, ABL1, ATM, AKT1, MAPK1, MAPK3, TRRAP, MAPK14, GSK3B, CSNK2A1, MAPK8, ATR, TAF1, HIPK2, TRRAP, PRLDC, CSNK2A2, RPS6KA2, CD7, and RPS6KA1. Drugs which are currently available for targeting the above kinases are given in Table 1.

**Table 1:** Potentially useful drugs to target invasive cervical cancer based on in silico pathway analysis

Target	Details	Possible drugs
ABL1	Abelson murine leukemia vitral homolog 1, protooncogene, non-receptor tyrosine kinase, expression regulated by miRNA-203, regulated by CDC -2 mediated phosphorylation suggesting a role in cell cycle regulation	Ponatinib – Dasatinib – Imatinib Nilotinib – Bafetinib – Bosutini - AT9283 - XL 228
AKT1	V-Akt murine thymoma viral oncogene homolog 1: enzyme that belongs to the AKT family of serine/threonine kinases, activated through Pi3K supressing apoptosis	Cenisertib – Ipasertib – Afuresertib – Uprosertib - AT13148 - AZD5363 - BAY1125976 - MK2206
ATM	Ataxia telangiectasia mutated gene: serotine/theronien protein kinase activated by double strand breaks, phosphorytlates several key proteins that initiate DNA damage check-point, leading to cell cycle arrest, DNA repair and apoptosis	Olaparib – Veliparib - KU55933 - KU60019 - CP466722 - CGK73 – Wortmannin - LY294002
ATR	Ataxia telangiectasia and Rad3 related: belongs to the PI3K family, serotine/theronien protein kinase activated by double strand breaks, phosphorytlates several key proteins that initiate DNA damage check-point, leading to cell cycle arrest, DNA repair and apoptosis	**ATR Inhibitors:**Schisandrin B - NU6027 – Dactolisib - VE-821 - VE-822 (VX-970) - AZ20 - AZD6738 - CGK73**PI3K** Copanlisib – Dactolisib – Buparlisib Pictilisib – Apitolisib – Omipalisib – Gedatolisib – Pilaralisib – Voxtalisib - BGT226 - NVP-BGT226 - GSK2636771 - PF-4691502 - PI-103 - SF1126
CSNK2A1/CSNK2A2:	Casein kinase 2 alpha 1/2 polypeptide: a serine/threonine protein kinase that phosphorylates acidic proteins/enzyme interacting with various substrates	CX-4945
TRRAP	Transforming/transcription domain associated protein: adapter protein, involved in epigenetic transcription activation, required for MYC, TO53 and EF2 mediated transcription activation, requied for mitotic check-point and cell cycle progression	**(c-MYC)**10058-F410074-G5 - 10074-A4**EF2** MOC31-PE
HIPK2	Homeodomain interacting protein kinase 2: regulates TGF-beta induced jun activation	A64
MAPK1/3	Mitogen activated protein kinase 1: member of the MAP kinase family, acts in a signallig cascade regulating cellc cyle, proliferation and differentiation	BVD-523 – Ralimetinib - MK-8353 - SCH900353 - LY2228820
MAPK14	Encodes an p38 alpha mitogen activated protein kinase, involved in several cellular functions varying from gene expression to programmed cell death	Losmapimod
RPS6KA1	Ribosomal protein S Kinase polypeptide 1: phosphorylates various substrates such as the MAPK family members, implicated in celle growth and differentation	SL-0101

### Biomarker discovery for early discovery

**Figure 11:**
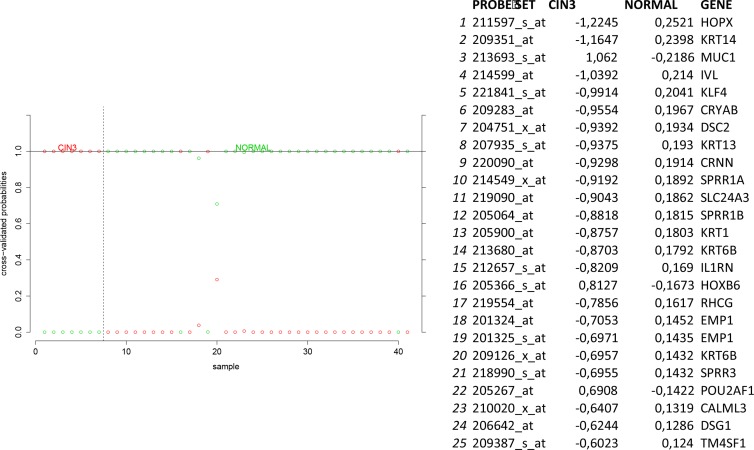
Biomarker discovery prediction analysis of Affymetrix microarrays comparing normal samples versus CIN III samples in GSE5787, GSE7803 and GSE9750

**Figure 12:**
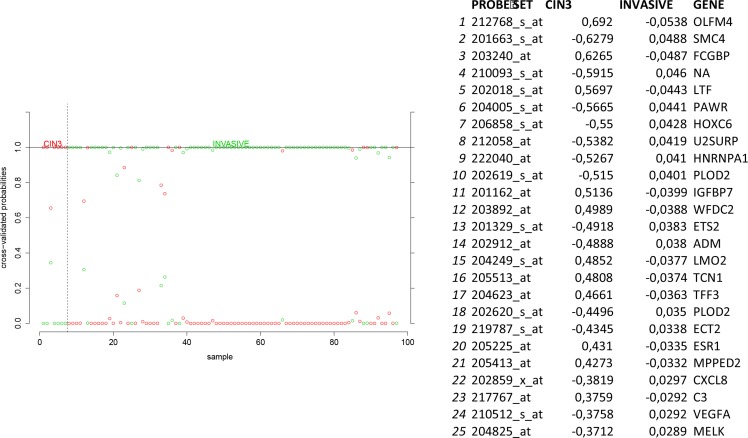
Biomarker discovery prediction analysis of Affymetrix microarrays comparing CIN III lesions versus invasive cervical cancer samples in GSE5787, GSE7803 and GSE9750

**Figure 13:**
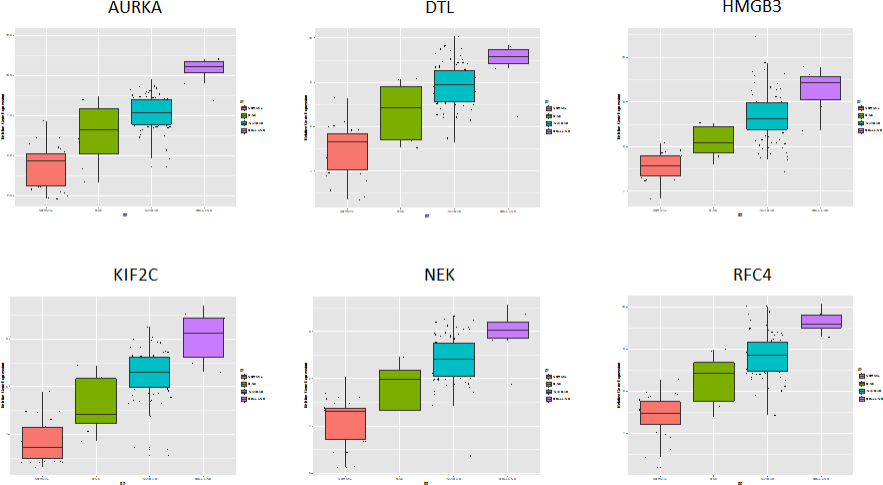
Biomarker Discovery [Prediction Analysis of Microarrays; Normal samples (red) vs. CIN III samples (green), Invasive cervical cancer samples (blue) vs. cell lines (purple)]

In order to identify biomarkers for early discovery, we adopted a two stage approach. First we used PAM to compare the expression profiles of normal and CIN3 samples as well as the expression profiles of CIN3 and invasive cancer samples. We identified 148 probe sets discriminating between normal and CIN3 samples (sensitivity = 100%, specificity = 91%, accuracy = 93%) and 334 probes sets discriminating between CIN3 cancer samples (sensitivity = 84%, specificity = 100%, accuracy = 85%). Data are shown in Figures 11 and 12 respectively. Of note, VEGFA was identified amongst the top 25 most discriminating biomarkers between CIN3 and cancer samples. We then looked for probe sets identified in both analyses and showing both increased expression in CIN3 samples respective to normal samples and in cancer samples respective to CIN3 samples. Adhering to these criteria, no probe sets were retained. Moreover, since this analysis is essentially intended to identify expression signatures and the classification strength of these signatures depends on the combined rather than the individual expression levels of the constituting genes, the presented methodology is not optimal for the identification of individual biomarkers. Therefore, we also adopted a second approach. We compared the lists of differentially expressed genes between normal and CIN3 samples on the one hand (*N* = 1,923) and between CIN3 and invasive cancer samples on the other hand (*N* = 628). We identified 7 probe sets that were significantly overexpressed (FC > 2 and FDR < 10%) in both CIN3 samples respective to normal samples and in cancer samples respective to CIN3 samples. From these, 6 probes set corresponding to 6 unique genes (i.e. AURKA, DTL, HMGB3, KIF2C, NEK2, and RFC4) were additionally overexpressed in cervical cancer cell lines respective to the cancer samples, suggesting they are cancer cell intrinsic and thus can be considered as potential biomarkers for cervical cancer tailored to early diagnosis. Expression levels are represented in Figure 13.

## DISCUSSION

Epidemiologic and experimental studies have shown that a persistent infection with a high risk HPV is the causal factor in the development of cervical cancer. [2]. A significant event in HPV-associated carcinogenesis is the induction of genomic instability and global disruption of cell gene expression, principally by the HPV E6 and E7 oncoproteins. Integration of HPV DNA into the host genome may confer growth and survival advantage via enhanced expression of viral oncoproteins, alteration of critical cellular genes, loss of function of DNA repair genes and tumor suppressor genes, and changes in global promotor methylation and transcription or microRNA expression [10, 11].

Previous studies have implicated somatic mutations in PIK3CA (14%), PTEN (6%), TP53 (5–27%), STK11 (4%) and KRAS (8%) as well as several copy number alterations in the pathogenesis of cervical carcinomas [3, 4]. Ojesnina et al. (2014) performed a whole exome sequencing analysis of 115 cervical carcinomas-normal paired samples, transcriptome sequencing of 79 cases and whole genome sequencing of 14 tumor-normal pairs. They found novel somatic mutations in the MAPK1 gene (8%), HLA-B gene (9%), EP300 (16%), FBXW7 (15%), NFE2L2 (4%), and ERBB2 (6%) of 79 squamous carcinomas, and ELF3 (13%) and CBFB (8%) mutations in 24 adenocarcinomas. Gene expression levels at HPV integration sites were significantly higher in tumors with HPV integration compared with expression of the same genes in tumors without viral integration at the same site. Single gene mutations are an important cause of pathway disruption, but there are many other factors implicated in pathway (in) activation (such as altered gene expression, gene interactions, effects of miRNAs, methylation, etc.) that may be of functional importance. We therefore used a different approach, gene set enrichment and pathway analysis, in an attempt to unravel the major driving signaling networks that are common in most cervical cancer patients and could be drugable.

A central challenge in analyzing genomic changes in cancer is distinguishing drivers from coincidental passengers [12] Possible approaches are to assume that passenger mutations are randomly distributed and to incorporate mutational heterogeneity into the analysis [13]. We have tried to reliably identify important driving pathways using a big sample size and only retaining genes for the analysis that were also abnormally expressed in the cell lines. Comparative analysis of different microarray types has shown to generate comprehensive and reliable results, provided that the data are normalized [14]. Tamborero et al. analyzed somatic mutations generated by all projects within Pan-Cancer [14]. This enabled them to make a comprehensive and reliable list of cancer driver genes, which contains most of the genes that play a prominent role in our analysis. Bignell et al. (2010) identified 2428 somatic homozygous deletions (HD) in 746 cancer cell lines. Fourteen HD were located over the known recessive cancer genes CDKN2A, PTEN, RB, SMAD4, NF1, MAP2K4, MSH2, TP53, NF2, MLH1, SMARCB1, PIK3R1, BMPR1A and CDH1, which also showed to be important in our analysis.

We identified a PPI-network consisting of 5 signaling modules. These ware associated with MYC signaling (Module 1), cell cycle deregulation (Module 2), TGFβ-signaling (Module 3), MAPK signaling (Module 4) and chromatin modeling (Module 5). C-MYC signaling is deregulated in many tumor types [15] and C-MYC amplification is well documented in cervical cancer [16]. Several studies show that C-MYC is a hot spot for HPV integration in the host cervical genome, suggesting that it may play an important role in cervical oncogenesis [17] The C-MYC oncogene is a “master regulator” controlling cellular growth regulation and cellular metabolism (eg. glycolysis, mitochondrial biogenesis, glutamine metabolism) [18]. The effect of C-MYC can occur either as a primary oncogene which is activated by amplification or translocation, or as a downstream effect of other activated oncogenes [18]. Kim et al. showed that C-MYC amplification is an independent prognostic marker for shorter disease free and cancer specific survival in cervical cancer treated by radical hysterectomy [16]. C-MYC is an attractive therapeutic target and therefore small molecules and antisense oligonuclotides have been developed in an attempt to downregulate C-MYC function [18] Other interesting partners in this module are signaling events mediated by histone deacetylases (HDAC) and E2F. HDAC remove the acetyl groups from the lysine residues leading to the formation of a condensed and transcriptionally silenced chromatin [19]. HDAC inhibitors are a new class of cytostatic agents that inhibit the proliferation of tumor cells in culture and *in vivo* by inducing cell cycle arrest, differentiation and apoptosis and may be of potential use in the treatment of cervical cancer [20]. The E2F transcription factor network consists of a family of transcription regulators [21]. Among E2F transcriptional targets are cyclins, CDKs, checkpoint regulators, DNA repair and replication proteins. Peptides inhibiting E2F transcription have recently been developed and showed antitumoral activity *in vitro* and in xenografts [22].

The cell cycle deregulation module contains several targetable components such as cyclins, checkpoint regulators and p53. There is renewed interest to develop drugs to restore p53 activity in patients with mutant or dysfunctional p53 as this is implicated in many tumors [23]. Loss of RB1 function, which is a well known phenomenon in cervical cancer leads to expression of Cyclin D1, over-expression/amplification of CDK4, and/or loss of the CDKN1B, CDKN2A and CDKN2B. Cervical tumors are therefore likely to be sensitive to CDK4/6 inhibitors. ATR, a kinase which phosphorylates p53 and other proteins such as CHK1 and RAD1, is an example of a novel cell cycle driver candidate which came up in our analysis, but also the study of Tamberero et al. on drivers in 12 different tumor types (not including cervical cancer) [14].

The TGFβ signaling module consists, besides the TGFβ–pathway, of related pathways regulating SMAD2/3 signaling, HIF-1 alpha transcription, coregulation of androgen receptor activity, FOXA1 transcription and some pathways related to viral infections. TGFβ is a multipotent cytokine that is involved in many cellular processes. Its action is context dependent. In the normal cell TGFβ induces apoptosis and controls differentiation and proliferation. During the early stages of carcinogenesis it inhibits proliferation of transformed tumor cells, but during tumor progression it supports tumor growth and enhances invasion and metastasis, tumor angiogenesis and systemic and local tumor suppression. Disruption of the TGFβ signaling pathway and diminished SMAD2 phosporylation are well documented in HPV16-immortalized human keratinocytes [24] and human cervical cancers. A recent study demonstrated that the genetic variation in immune-related genes is associated with the susceptibility to HPV related cancers and implicates TGFβR1/TGFβ signaling early in the development of both oropharyngal and cervical cancer [25]. A study of Ki et al. suggests that alteration of TGF-βRII, SMAD2 and SMAD4 may play an important role the development and progression of cervical carcinomas [26]. Kloth et al. showed that weak cytoplasmic SMAD4 expression and absent nuclear SMAD4 expression were significantly correlated with poor-disease-free and overall 5-year survival in an immunohistochemical study on 117 primary cervical carcinomas, suggesting that SMAD4 is a target molecule for functional inactivation in cervical cancer [27] Blockade of TGFβ and its signaling pathway provide multiple therapeutic opportunities, which has lead to the development of TGFβ antibodies, antisense oligonucleotides and receptor kinase inhibitors [28]. The hypoxia inducible transcription factor (HIF) coordinates the response of tumors to low oxygen by stimulating genes involved in metabolism and angiogenesis. Up to 70% of cervical carcinomas are expressing moderate to high levels of HIF-1 [29]. Strong immunopositivity of HIF-1 has been associated with a bad prognosis in early cervical cancer, making HIF-1 inhibition an attractive treatment option. VEGF, a downstream target of HIF-1, plays a critical role in angiogenesis [30].

The MAP-kinase module contains many potential targets for treatment: RAS pathway, Toll-like receptors cascades, MAPK signaling pathway, ERBB2/ERBB3 signaling events, S1P2 pathway (including such as jun, fos, PI3K, MAPK1, MAPK14,…) and RAS signaling in the CD4+ TCR pathway. The RAS-MAPK and PI3K-mTOR signaling pathways are deregulated in many cancers by genetic and epigenetic aberrations [31]. Several key components in these pathways, such as RAS, B-RAF, C-RAF, MEK1, PI3Kand AKT are activated by mutations or gene amplifications, while other components that inhibit these pathways, such as PTEN, LBK1 and TSC1/2, are inactivated by genomic deletions and mutations. In addition alternative splicing can affect the activity of signaling effectors contributing to their constitutive or improper function. The most well characterized examples are represented by members of the receptor tyrosine kinase family (ERBB-2, EGFR, FGFR, INSR, VEGFR, MET and RON), the non-receptor cytosolic protein kinases (such as SRC, RAS, RAF) and non-kinase cytosolic recptors (such as androgen and estrogen receptors) [32]. Mutations in the PI3K CA gene, located at chromosome 3q24-29, are seen in up to 36% of cervical cancers [33]. An increased copy number is positively correlated with 3q26.3 amplification in both cervical tumor tissues and cancer cell lines. Quantitative RT-PCR analysis showed that the PI3K gene copy number is 3 or more in 28 out of 40 cases and that it triggered AKT phosphorylation in 39 out of 46 examined neoplastic tissues [34]. Schwarz et al. [35] showed by means of Affymetrix whole genome analysis that activation of the PI3K pathway in patients with cervical cancer was associated with an incomplete metabolic response to chemoradiation and should be considered as an important novel therapeutic target [36]. Notch signaling seems to play an important role during cervical carcinogenesis as it activates the PI3K/AKT pathway and upregulates C-MYC. Coactivation of Notch 1 and NF-kB signaling pathways at the cellular level is seen can be found in the majority of human cervical cancers [37].

Chromatin remodeling plays a central role in the regulation of gene expression by providing the transcription machinery with dynamic access to an otherwise tightly packed genome [38]. Deregulation of chromatin remodeling causes loss of transcriptional regulation at critical check-points required for proper cellular functions, such as the tumor suppressor RB protein functions. Shadeo et al. [39] showed that impaired regulation of chromatin remodeling complex components occurs in the development of CIN lesions. TRRAP (Transformation/transcription domain associated protein) plays a central role in this module. TRRAP overexpression or stabilization is known to induce multiple mitotic defects, including lagging of chromosomes, chromosome bridges and multipolar spindles [40].

The findings of the current pathway analysis are in line with the results of other genome wide studies in cervical cancer cell lines and human samples. Higerda-Almaraz et al. performed an in silico overexpression analysis of transcription factors in six cervical cancer cell lines using a hypergeometric test and employing the SwitchGear Database for the acquisition of transcription start sites [41]. They found that the transcription factors E2F1, TCF4, C-MYC, MAX, E2F6 and NFKB were most significantly overexpressed. Perez-Plasencia used Human Whole Genome Codelink microarrays to analyze 8 HPV 16 squamous cervical carcinoma and 3 normal cervical samples [42]. Cervical carcinoma proved to be associated with important upregulation of Wnt signaling pathway, which was validated by *in situ* hybridization. In their analysis important other upregulated pathways were those of calcium signaling and MAPK signaling, as well as cell cycle-related genes. There was down regulation of focal adhesion, TGFβ signaling and other metabolic pathways. Mine et al. generated gene expression data from 40 cervical tumors and 20 normal tissue samples. Combining these with data from 4 other independent studies they could reveal a robust set of differentially expressed genes and used it to reconstruct a gene co-expression network, using a different methodology then in our paper. They could identify three sub-networks: one containing genes involved in the cell cycle, another related to antiviral activity, and a third minor network related to epithelial differentiation. Both the cell cycle and antiviral sub-networks were upregulated in cervical cancers. From the cell cycle subnetwork six genes(CEP70, GMPS, CM2, NAT13, RFC4 and TOPBP1) located in regions of frequent chromosomal aberrations were identified. The finding of increased expression of genes related to the antiviral response was surprising. Among these genes there were innate immune sensors of viruses (ADAR, AIM2), molecules involved in antigen presentation (HLA, TAP, RFX5), transcription regulators (IRF1, IRF7, IRF9 and STAT1) and oncogenes of innate immunity directly involved in the elimination of viruses (such as HERC5, MX1, OAS2, ISG15 and RSAD2) [43].

In the present paper we identified six putative biomarkers (AURKA, DTL, HMGB3, KIF2C, NEK2, and RFC4) which should be further tested to separate indolent HPV related cervical infections from progressive CIN III lesions and for early diagnosis of cervical cancer. The potential targets for systemic treatment of advanced cervical cancer identified in this study should be considered as hypothesis raising. Further detailed *in vitro* and *in vivo* studies, linking genotype to phenotypes, are necessary to explore the effectivity of manipulating the potentially interesting pathways we proposed in this paper. Better knowledge on how to detect the crucial elements in activated or suppressed pathways, and correcting abnormal signaling beyond the mutational level is needed for the successful rational targeting cervical cancer. Several questions remain: which and how many pathways do we have to manipulate to reverse the malignant phenotype, do we have to focus on mutations or could we just look at disrupted signaling, what are the effects of tumor heterogeneity and tumor progression? As no information was available for the HPV status of all samples we were not able to assess differences in gene expression between HPV positive and HPV negative cervical carcinoma. Although 90–95% of cervical carcinomas are HPV positive, theoretically this may be important as the biology of cervical cancer may be different in both groups. The data we presented regard squamous carcinoma of the cervix as the current series included only one invasive adenocarcinoma. A limitation of this study is that only gene expression data were used. A complete integration of genomic and proteomic data, as was done in The Cancer Genome Atlas (TCGA) project for 13 other tumor types, should be considered for cervical cancer in order to further improve our understanding of the disease [44].

## MATERIALS AND METHODS

### Patient data sets

**Figure 14:**
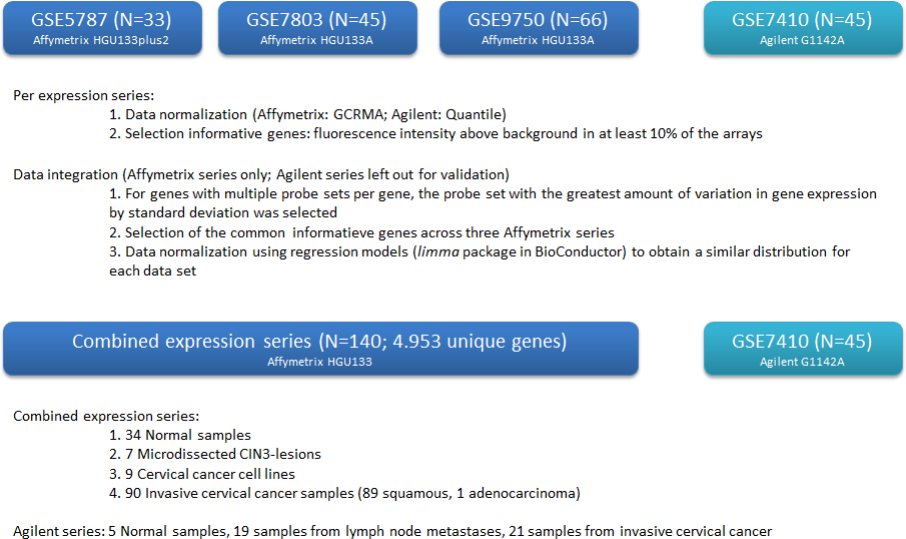
Overview of study design

All publicly available Affymetrix data sets (HGU133-series) containing normal and pretreatment (pre)invasive cervical cancer samples with relevant clinical information were retrieved from the Gene Expression Omnibus (GEO; http://www.ncbi.nlm.nih.gov/gds): GSE5787, GSE7803 and GSE9750. Combined, these data sets vouch for a total of 140 samples including 34 normal samples, 7 micro-dissected CIN3 lesions, 9 cervical cancer cell lines (C4-1, CaSki, C-33A, HT-3, SiHA, SW756, MS751, ME-180, HeLa) and 90 invasive cervical cancer samples. In addition, the only available Agilent data set fulfilling the same criteria (GSE7410; 5 normal cervical samples, 35 samples from invasive cervical cancer) was selected as a validation set (Figure 14).

### Data normalization and exploration

Expression series were normalized using GCRMA for the Affimetrix platforms and Quantile normalization for the Agilent data [45]. Informative genes were selected if they had a fluorescence intensity above background in at least 10% of the arrays. Next the data were integrated for the Affimetrix series only, leaving out the Agilent series for validation. For genes with multiple probe sets per gene, the probe set with the greatest amount of variation in gene expression by standard deviation was selected. Then common informative genes were selected across the three Affymetrix series. The data were further normalized using regression models (limma package in Bioconductor) to obtain similar distributions for each data set, as described previously [45]. Unsupervised hierarchical cluster analysis (UHCA) was performed on the integrated data set using the 500 most variable probe sets only. Sample dissimilarity was calculated using the Manhattan distance and the dissimilarity matrix was clustered using Ward-linkage. Annotated heatmaps were generated using the BioConductor-package *Heatplus* in R. Cluster robustness analysis was performed using the silhouette score on 10 randomly permuted subseries (*N* = 120) as described before [45]. To identify probe sets specifically upregulated in cervical cancer samples, we adopted a three-stage analysis strategy: A. We compared the expression profiles of normal cervix samples to samples from cervical cancer; B. Probe sets overexpressed in cervical cancer samples were compared between the same cervical cancer samples and cervical cancer cell lines to assess cancer cell-specific expression; and C. Probe sets overexpressed in the cervical cancer cell lines relative to the cervical cancer samples were validated in the Agilent series. For each stage, differential expression for the relevant genes was assessed using linear regression models and *P*-values were adjusted for false discovery using the Benjamini and Hochberg correction. Corrected *P*-values less than 0.05 were considered significant.

### Analysis of pathway and biological processes

Validated genes were interrogated using gene set enrichment analysis (GSEA; http://amp.pharm.mssm.edu/Enrichr/enrich) to unravel cervical cancer biology and using Expression2Kinases (E2K) to delineate the driving signaling network, For GSEA, gene sets associated with pathways in the KEGG database and with gene ontologies related to biological processes were tested. Gene sets with an FDR-corrected *P*-value less than 10% were considered significant. The signalling network identified through E2K was further analyzed using the Reactome FI-plugin in CytoScape version 3. First, spectral clustering was performed to identify the signalling modules within the network. Then, each signalling module was analyzed for enriched pathways. Correlations were calculated with the Pearson correlation methods in SPSS 16.0 statistical software packages. Standard errors for Pearson correlation coefficients were estimated by the formula SE (1-Rho^2)/SQRT(n-1) Cox proportional regression models estimated survival hazard ratios withn 95% confidence intervals. Meta-analysis was done using MIX 2.0 software using a random effect model for relative risk and correlation coefficients.

### Biomarker analysis for early diagnosis

In order to detect potential biomarkers for early diagnosis, we performed predication analysis of microarrays comparing normal cervical samples to CIN3 samples and CIN3 samples to invasive cervical cancer samples. Using ten fold cross-validation, a delta value was chosen that minimizes the misclassification error rate. The delta value relates to the expression difference between two classes that is considered relevant for probe sets to be included in the classifiers. Thus, using the delta-value not only the misclassification error rate is minimized but also the relevant probe sets are selected. We then compared the resulting classifiers to identify probe sets with low, intermediate and high expression levels in respectively normal, CIN3 and cancer samples. In addition, we also compared the lists of differentially expressed probe sets between normal and CIN3 samples on the one hand and between CIN3 and cancer samples on the other hand. Probe sets with a significant (FDR < 5%) 2-fold increased expression level in CIN3 samples in contrast to normal samples, in cancer samples in contrast to CIN3 samples and in cervical cancer cell lines in contrast to cancer samples were retained.
